# Association of cumulative oxygen and carbon dioxide levels with neurologic outcome after pediatric cardiac arrest resuscitation: A multicenter cohort study

**DOI:** 10.1016/j.resplu.2024.100804

**Published:** 2024-10-24

**Authors:** Marijn Albrecht, Rogier C.J. de Jonge, Jimena Del Castillo, Andrea Christoff, Matthijs De Hoog, Sangmo Je, Vinay M. Nadkarni, Dana E. Niles, Oliver Tegg, Kari Wellnitz, Corinne M.P. Buysse, for the pediRES-Q Collaborative Investigators

**Affiliations:** a Department of Neonatal and Pediatric Intensive Care, Division of Pediatric Intensive Care, Erasmus MC Sophia Children’s Hospital, Rotterdam, the Netherlands; b Pediatric Intensive Care Department, Hospital General Universitario Gregorio Marañón, Instituto de Investigación Sanitaria Gregorio Marañón, Madrid, Spain; c Pediatric Intensive Care Unit, The Children’s Hospital at Westmead, Sydney, Australia; d Center for Simulation, Innovation, and Advanced Education, Children’s Hospital of Philadelphia, PA, United States; e Department of Anesthesiology and Critical Care, The Children’s Hospital of Philadelphia, Philadelphia, PA, United States; f Department of Anesthesiology and Critical Care, University of Pennsylvania Perelman School of Medicine, Philadelphia, PA, United States; g Division of Pediatric Critical Care, Stead Family Department of Pediatrics, University of Iowa Carver College of Medicine, Iowa City, Iowa, United States

**Keywords:** Pediatric cardiac arrest, Post-cardiac arrest care, Neurologic outcome, Ventilation, Oxygenation

## Abstract

**Objective:**

We aimed to (1) determine the association between cumulative PaO_2_ and PaCO_2_ exposure 24 h post-return of circulation and survival with favorable neurologic outcome. And (2) to assess adherence to American Heart Association post-cardiac arrest care treatment goals (PaO_2_ 75–100 mmHg and PaCO_2_ 35–45 mmHg).

**Design and setting:**

Prospectively collected data were analysed from five Pediatric Resuscitation Quality collaborative sites supplemented with retrospective PaO_2_ and PaCO_2_ data.

**Patients:**

Children aged 1 day–17 years with return of circulation after cardiac arrest, admitted 2019–2022, with ≥ 4 arterial blood gasses spanning at least 12 h within 24 h post-return of ciculation, were eligible. Congenital cyanotic heart disease events were excluded.

**Measurements:**

Area under the curve calculation provided hourly cumulative PaO_2_ and PaCO_2_ exposures per child and similarly guideline recommended cumulative ranges. The primary outcome was survival to hospital discharge with favorable neurologic outcome defined as a Pediatric Cerebral Performance Category 1–3, or no pre-arrest baseline difference.

**Main results:**

Among 292 included children (median age 2.6 years (IQR 0.4–10.9)), 57 % survived to discharge and 48 % had favorable neurologic outcome (88 % of survivors). Cumulative PaO_2_ and PaCO_2_ exposure 0–24 h post-return of circulation were not significantly associated with favorable neurologic outcome in multivariable analysis (OR 1.0, 95 %CI 0.98–1.02 and OR 0.97, 95 %CI 0.87–1.09 respectively). Cumulative PaO_2_ and PaCO_2_ remained within guideline recommended ranges for 24 % and 58 % of children respectively with median areas under the curve over 0 – 24 h of 2664 mmHg (2151 – 3249 mmHg) for PaO_2_ and 948 mmHg (853 – 1051 mmHg) for PaCO_2_. AHA post-cardiac arrest care guideline recommendations for PaO_2_ (1800–2400 mmHg) and PaCO_2_ (840–1080 mmHg) were recalculated as area under the curve ranges. Achieving both normoxia and normocapnia was observed in 12 % of children.

**Conclusions:**

Cumulative PaO_2_ and PaCO_2_ exposure in the first 24 h post-return of circulation was not associated with survival with favorable neurologic outcome. Pediatric AHA post-cardiac arrest care guideline normoxia and normocapnia goals were often not met. Larger cohort studies are necessary to improve the accuracy of cumulative exposure calculations, assess the long-term effects of PaO_2_ and PaCO_2_ exposure, and explore the influence of other post-cardiac arrest care therapeutic strategies.

## Introduction

Survival to hospital discharge rates after in- and out-of-hospital pediatric cardiac arrest (pIHCA and pOHCA) remain low 1, 2, 3, 4, 5, 6. Apart from rapid recognition and high-quality (bystander) CPR, preventing ischemia–reperfusion injury improves outcome after arrest ^7^. However, oxygenation and ventilation post-arrest can be challenging ^8^. The current American Heart Association post-cardiac arrest care (PCAC) guideline recommends titrating to achieve normal PaO_2_ (75–100 mmHg) and PaCO_2_ (35–45 mmHg) levels respectively ^8^.

Despite the known risks of hypoxemia, the evidence for the post-cardiac arrest care recommendations is scant and based on studies using cutoff values 2, 9, 10, 11. Summarized in a systematic review, three single-centre cohorts did not find any association with oxygen exposure (cutoff > 300 mmHg) and survival to hospital discharge 9, 10, 11. In the only pediatric study on hypocapnia and hypercapnia (cutoff PaCO_2_ < 30 and > 50 mmHg), both were shown to be associated with worse survival to hospital discharge ^11^.

Cumulative exposure using area under the curve calculation (AUC) for O_2_ and CO_2_ may provide a more comprehensive representation of their physiological dynamics, as suggested by Van Zellem ^12^. In this multicentre prospective cohort study, we aim to determine the association of cumulative PaO_2_ and PaCO_2_ exposure 24 h after pediatric cardiac arrest resuscitation with neurologic outcome, and to assess post-cardiac arrest care treatment goals adherence. We hypothesized a U-shaped association between cumulative PaO_2_ and PaCO_2_ levels and unfavorable outcomes, suggesting that high and low extremes may be linked to adverse clinical outcomes.

## Methods

### Study design

Five hospitals in an international prospective multicentre Pediatric Resuscitation Quality partnership (pediRES-Q) (ClinicalTrials.gov: NCT02708134, Appendix 1) participated in this study. The study was approved by each hospital’s research ethics board (please see the supplemental digital content for more information) and procedures were followed in accordance with the ethical standards of the responsible committee on human experimentation and with the Helsinki Declaration of 1975 (as most recently amended). Consent waiver was obtained per United States Code of Federal Regulations 45 CFR 46.116(d) and 45 CFR 46.408(a) in compliance with the Health Insurance Portability and Accountability Act.

### Inclusion criteria

All children with ROC after IHCA or OHCA aged > 1 day and < 18 years admitted to one of the study hospital pediatric intensive care units (PICUs) between January 2019 and February 2022 were eligible. Cardiac arrest was defined as ≥ one minute of cardiopulmonary resuscitation (CPR). Arrests in neonates < 24 h old (perinatal asphyxia), children with congenital cyanotic heart disease and events with < 4 arterial blood gas (ABG) samples taken within the first 24 h post-ROC or any number of ABGs spanning less than 12 h were excluded.

### Data collection

The pre-, intra- and post-arrest variables are prospectively collected for each cardiac arrest event within pediRES-Q. PaO_2_ and PaCO_2_ data were collected retrospectively based on predefined standard criteria, without blinding to outcomes. Data were derived from ambulance registration forms, Patient Data Management Systems (PDMS), and cardiac arrest registration forms. Pre-arrest variables included: gender, age at time of event, and pre-arrest pediatric cerebral category scale (PCPC). Intra-arrest variables included: location of arrest (in-hospital or out-of-hospital), bystander CPR (yes or no), initial rhythm (shockable, non-shockable, or unknown), CPR duration, aetiology of event (medical non-cardiac, medical cardiac, surgical non-cardiac, surgical cardiac, trauma, or unknown) and extracorporeal CPR (ECPR). Post-arrest variables included: post-ROSC extracorporeal membrane oxygenation (ECMO), re-arrest within the same admission, laboratory values and outcome (survival to and neurologic outcome at hospital discharge). Collected laboratory values were arterial pH, lactate, PaO_2_ and PaCO_2_ (presented as cumulative exposure and by cutoff based on existing literature: PaO_2_ < 60/>200/>250/300 mmHg and PaCO_2_ < 30/>50 mmHg) ^13^.

### Outcome measures

The primary outcome measure was survival to hospital discharge with favorable neurologic outcome defined as a PCPC of 1 to 3, or no pre-arrest baseline difference ^14^. The secondary outcome measures were 1. survival to hospital discharge and 2. favorable neurologic outcome only in the survivors (i.e. excluding PCPC 6). The research questions were: 1) to determine the association between cumulative exposure of PaO_2_ and PaCO_2_ with outcome, and 2) to assess adherence to AHA post-cardiac arrest care treatment goals of normoxia and normocapnia.

### Statistical analysis

Baseline characteristics and survival outcome were reported using descriptive statistics. Categorical variables were reported as frequencies (n) and percentages (%). Continuous variables were reported as medians with first and third quartile (Q1;Q3). Differences between groups were tested using Fisher’s exact test for categorical variables and Mann-Whitney *U* test for continuous variables.

Cumulative exposure to PaO_2_ and PaCO_2_ was calculated using the area under the curve (AUC) for each included child. The trapezoidal method was applied with *T = 0* representing the time of ROC, by dividing the total area under the curve into smaller segments—such as squares, rectangles, and triangles. The areas of these shapes were then calculated using standard geometric formulas and combined to determine the total area under the curve. ^15^. All available ABGs per child were divided into two time intervals: 0–6 h and 7–24 h post-ROC, chosen to capture the early phase of the post-cardiac arrest syndrome ^8^. Two AUCs were calculated per child based on the ABGs that approximated these time intervals. To standardize these cumulative values and enable comparison across patients, the AUCs were divided by the time covered by each child's ABGs within the two epochs and multiplied by 6 h for the 0–6 h interval and 18 h for the 7–24 h interval, and combined into a 24 h interval resulting in three time intervals (0–6, 7–24 and 0–24 h) ^12^. Prior to analysis, cumulative PaO2 and PaCO2 values were rescaled by dividing by 100 in order to keep them in a similar range to other variables in the multivariable analysis. Guideline recommendations (PaO_2_ 75–100 mmHg and PaCO_2_ 35–45 mmHg respectively) were calculated into AUCs likewise for the three time intervals.

Differences over the three time intervals between the median of the means of the cumulative exposures and the medians of the recalculated AUC guideline recommendations, were tested with a Wilcoxon signed-rank test. Univariable and multivariable logistic regression was used thereafter to investigate the association between cumulative PaO_2_ and PaCO_2_ exposure over the three time-intervals, and survival to discharge with a favorable neurologic outcome. Secondary analyses were performed for 1. survival and 2. neurologic outcome in survivors (i.e. PCPC 4 and 5). Preselected variables for the multivariable model included: age, gender, location, rhythm, aetiology, lowest pH and highest lactate. A correlation matrix using a cutoff of > 0.7 was used for variable exclusion. Odds ratio’s and 95 %-confidence intervals (CI) were reported. Sensitivity analyses were performed for favorable outcome definition (a PCPC of 1–2 instead of 1–3), age groups (infant < 1 year old, child 1–11 years old, adolescent 12–17 years old) and event location (in-hospital or out-of-hospital). Statistical significance was considered based on a two-tailed p-level < 0.05. All analyses were conducted using Statistical Package of Social Sciences software (IBM Corp. Released 2021. IBM SPSS Statistics for Windows, Version 28.0. Armonk, New York).

## Results

### Child and cardiac arrest characteristics

**Fig. 1:**
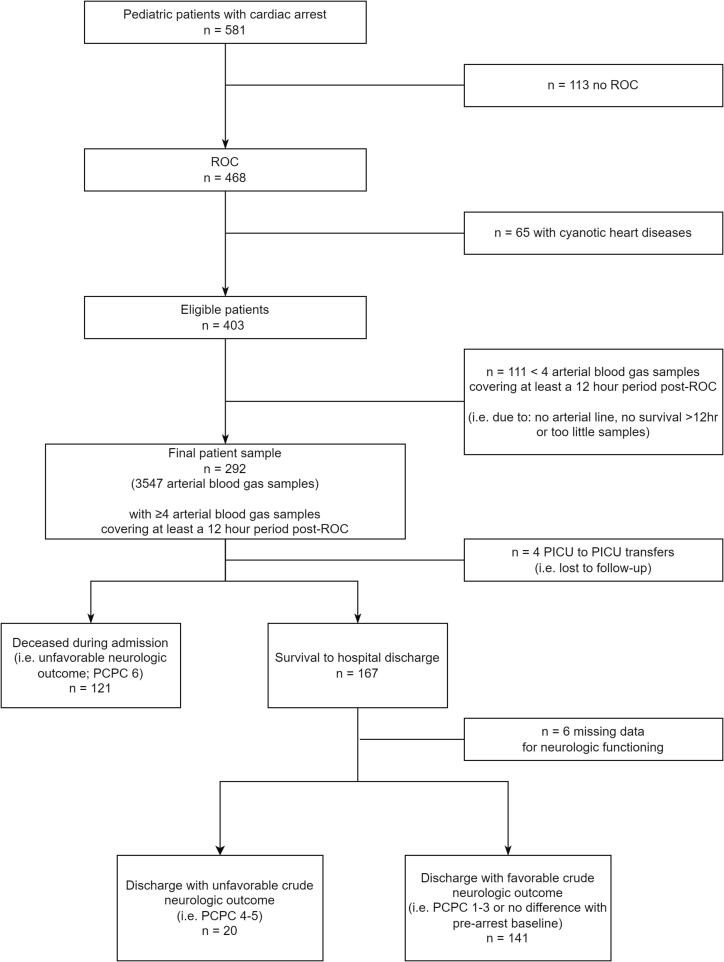
Overview of selection and main outcomes. Abbreviations: ROC = Return of Circulation, PICU = Pediatric Intensive Care Unit, PCPC = Pediatric Cerebral Performance Category.

An overview of the inclusion is depicted in Fig. 1 and the basic characteristics are presented in Table 1. During the inclusion period, 581 events were recorded. Of those, 468 achieved ROC (81 %). A total of 176 children were excluded: 65 because of cyanotic congenital heart disease and 111 because of insufficient ABGs. Of the final sample of 292 children, four were lost to follow-up due to PICU-to-PICU transfers and six because information on neurologic functioning at discharge was missing.

**Table 1:** Child, cardiac arrest, arterial blood gas sample and outcome characteristics.

Characteristics including the number of missing values if applicable	Overall n^a^ = 292	Favorable neurologic outcome at hospital discharge n^a^ = 141	Non-favorable neurologic outcome at hospital discharge n^a^ = 141	p-value^d^
Age (years)^b^	2.6 (0.4–10.9)	1.8 (0.3–10.1)	3.5 (0.6–12.2)	0.080
Male gender^c^	168/292 (58)	82/141 (58)	80/141 (57)	0.904
Out-of-hospital cardiac arrest^c^	133/292 (46)	47/141 (33)	85/141 (60)	<0.001
Bystander BLS^c^ n = 4 missing	87/133 (65)	32/47 (68)	54/81 (67)	0.999
CPR duration (minutes)^b^ n = 2 missing	15.0 (5.0–33.0)	10.0 (5.0–20.0)	25.0 (10.0–45.0)	<0.001
Initial rhythm^c^				0.050
Shockable (VF/VT)	44/292 (15)	28/141 (20)	14/141 (10)	0.029
Non-shockable	195/292 (67)	91/141 (65)	98/141 (70)	0.447
Unknown/Not documented	53/292 (18)	22/141 (16)	29/141 (21)	0.353
Etiology category^c^				<0.001
Medical non cardiac	138/292 (47)	58/141 (41)	76/141 (54)	0.042
Medical cardiac	47/292 (16)	29/141 (21)	15/141 (11)	0.032
Surgical cardiac	49/292 (17)	34/141 (24)	14/141 (10)	0.002
Surgical non cardiac	12/292 (4)	8/141 (6)	3/141 (2)	0.217
Trauma	42/292 (14)	11/141 (8)	30/141 (21)	0.002
Unknown/Not documented	4/292 (1)	1/141 (1)	3/141 (2)	0.622
ECPR^c^	37/292 (13)	21/141 (15)	15/141 (11)	0.372
Post-ROSC ECMO^c^	27/292 (9)	19/141 (14)	8/141 (6)	0.041
Re-arrest^c^	16/292 (5)	4/141 (3)	11/141 (8)	0.109
Number of blood gases per child^b^	11.0 (8.0–15.0	10.0 (8.0–15.0)	13.0 (10.6–16.0)	0.002
Post-ROC time coverage per child before extrapolation (in hours)^b^	22.5 (21.1–23.3)	22.4 (21.1–23.2)	22.7 (21.1–23.3)	0.436
Time to first arterial blood gas after ROC (in hours)^b^	1.4 (0.4–3.6)	1.2 (0.3–3.3)	1.7 (0.5–3.7)	0.062
Lowest pH within 24 h after ROC^b^	7.18 (7.03–7.28)	7.23 (7.09–7.30)	7.12 (6.98–7.23)	<0.001
Highest lactate within 24 h after ROC (in mmol/L)^b^	6.3 (3.3–12.0)	5.0 (2.5–8.7)	10.1 (4.4–15.2)	<0.001
Min. PaO_2_ < 60 mmHg^c^	103/292 (35)	44/141 (31)	55/141 (39)	0.212
Max. PaO_2_ > 200 mmHg^c^	169/292 (58)	79/141 (56)	52/141 (37)	0.275
Max. PaO_2_ > 250 mmHg^c^	125/292 (43)	56/141 (40)	69/141 (49)	0.150
Max. PaO_2_ > 300 mmHg^c^	86/292 (29)	40/141 (28)	46/141 (33)	0.518
Min. PaCO_2_ < 30 mmHg^c^	102/292 (35)	36/141 (26)	65/141 (46	<0.001
Max. PaCO_2_ > 50 mmHg^c^	185/292 (63)	76/141 (54)	102/141 (72	<0.001
AUC PaO_2_ 0–––6 h mmHg^b^	666.7 (440.4–968.6)	694.7 (459.0–994.2)	624.1 (397.4–950.0)	0.232
AUC PaO_2_ 7–––24 h mmHg^b^	1920.5 (1608.3–2344.6)	1895.6 (1584.4–2233.1)	2037.8 (1667.1–2430.1)	0.065
AUC PaO_2_ 0–––24 h mmHg^b^	2663.6 (2150.6–3249.1)	2646.1 (2086.3–3254.6)	2715.5 (2253.3–3272.8)	0.380
AUC PaCO_2_ 0–––6 h mmHg^b^	214.8 (161.7–262.8)	225.2 (162.5–263.6)	207.6 (162.3–261.6)	0.522
AUC PaCO_2_ 7–––24 h mmHg^b^	736.9 (672.6–819.8)	736.3 (671.5–819.0	743.7 (673.2–825.8)	0.899
AUC PaCO_2_ 0–––24 h mmHg^b^	948.2 (853.3–1050.8)	945.7 (855.6–1064.0)	948.1 (849.5–1041.5)	0.584
Survival to hospital discharge^c^ n = 4 missing	167/288 (58)	141/141 (100)	20/141 (14)	<0.001
Favorable pre-arrest PCPC (PCPC 1–2)^c^ n = 1 missing	135/170 (79)	122/141 (87)	15/19 (79)	0.482

a Number of subjects in whom the variable was obtained unless otherwise specified.

b Median (Q1-Q3).

c Number of subjects/number of subject in whom the variable was obtained (percentage).

d p-value: independent sample *t*-test for continuous data or Mann-Whitney *U* test dependent on normality; Fisher’s exact test for dichotomous data. Abbreviations: BLS = Bystander Life Support, CPR = Cardiopulmonary resuscitation, VF = Ventricular fibrillation, VT = Ventricular tachycardia, ECPR = Extracorporeal Cardiopulmonary resuscitation, ECMO = Extracorporeal Membrane Oxygenation, ROSC = Return of Spontaneous Circulation, Pa = Partial pressure, mmHg = millimeters mercury, AUC = Area under the curve, PCPC = Pediatric Cerebral Performance Category.

**Table 2:** Univariable and multivariable logistic regression analysis of the primary outcome: all children with favorable (crude) neurologic outcome at discharge defined as survival with a PCPC of 1–3 or no difference with pre-arrest PCPC at discharge as dependent variables.

	Survival with post-arrest PCPC 1–3 or ΔPCPC 0 at hospital discharge			
AUC Variable	Crude		Adjusted^a^	
	OR (95 % CI)	p-value	OR (95 % CI)	p-value
AUC PaO_2_ 0–––6 h mmHg^b^	1.039 (0.958–1.096)	0.162	1.038 (0.974–1.105)	0.255
AUC PaO_2_ 6–––24 h mmHg^b^	0.989 (0.961–1.017)	0.425	0.989 (0.958–1.021)	0.508
AUC PaO_2_ 0–––24 h mmHg^b^	1.000 (0.980–1.021)	0.976	1.000 (0.977–1.023)	0.995
AUC PaCO_2_ 0–––6 h mmHg^b^	1.011 (0.760–1.344)	0.940	1.017 (0.687–1.504)	0.934
AUC PaCO_2_ 7–––24 h mmHg^b^	1.025 (0.904–1.161)	0.703	0.942 (0.818–1.085)	0.409
AUC PaCO_2_ 0–––24 h mmHg^b^	1.025 (0.934–1.126)	0.598	0.974 (0.872–1.088)	0.643

a Adjusted for: age at arrest, gender, location of arrest, rhythm, etiology of arrest, lowest pH within 24 h and highest lactate within 24 h.

b Value was rescaled by dividing by 100 in advance of the regression analysis.Abbreviations: CI = Confidence Interval, Min. = Minimum, Max. = Maximum, Pa = Partial pressure, mmHg = Millimeter mercury, AUC = Area under the curve.

The median age of included children was 2.6 years (Q1;Q3 0.4–10.9). Half of the population suffered an OHCA (46 %). A median of 11 blood gas samples were drawn per child covering 22.5 h post-ROC before extrapolation. During admission 121 children died (41 %), whereas 167 survived to hospital discharge. Of these 167 children, 141 (88 %) had favorable neurologic outcome.

### Association between cumulative PaO_2_ and PaCO_2_ exposure and outcome

Cumulative PaO_2_ and PaCO_2_ exposures over three time periods (0–6, 7–24 and 0–24 h) are presented in Table 3. Univariable and multivariable logistic regression showed no significant associations between cumulative PaO_2_ and PaCO_2_ exposure over three time intervals, and both favorable neurologic outcome at hospital discharge and survival to discharge alone (Table 2 and Supplemental Table S1). A potential U-shaped association was investigated (for the 0 – 24 h time interval) by examining the exposure variable across quartiles using regression analysis. However, no significant associations were found in both crude and adjusted analyses.

**Table 3:** Cumulative PaO2 and PaCO2 exposures 0–6, 7–24 and 0–24 h post-ROC of all children split by favorable neurologic outcome at discharge and compared to the guideline recommended normal range.

Oxygen and carbon dioxide exposure in three time epochs	Guideline recommended normal range^a^	Overall n^b^ = 292	Favorable neurologic outcome at hospital discharge n^b^ = 141	Non-favorable neurologic outcome at hospital discharge n^b^ = 141	p-valued1	p-valued2
0 – 6 h						
AUC PaO_2_ 0–––6 h mmHg^c^	450–––600	666.7 (440.4–968.6)	694.7 (459.0–994.2)	624.1 (397.4–950.0)	<0.001	<0.001
AUC PaCO_2_ 0–––6 h mmHg^c^	210–––270	214.8 (161.7–262.8)	225.2 (162.5–263.6)	207.6 (162.3–261.6)	<0.001	<0.001
						
7 – 24 h						
AUC PaO_2_ 7–––24 h mmHg^c^	1350–––1800	1920.5 (1608.3–2344.6)	1895.6 (1584.4–2233.1)	2037.8 (1667.1–2430.1)	<0.001	<0.001
AUC PaCO_2_ 7–––24 h mmHg^c^	630–––810	736.9 (672.6–819.8)	736.3 (671.5–819.0	743.7 (673.2–825.8)	0.991	0.819
						
0 – 24 h						
AUC PaO_2_ 0–––24 h mmHg^c^	1800–––2400	2663.6 (2150.6–3249.1)	2646.1 (2086.3–3254.6)	2715.5 (2253.3–3272.8)	<0.001	<0.001
AUC PaCO_2_ 0–––24 h mmHg^c^	840–––1080	948.2 (853.3–1050.8)	945.7 (855.6–1064.0)	948.1 (849.5–1041.5)	0.919	0.435

a Guideline recommendations (PaO_2_ 75–100 mmHg and PaCO_2_ 35–45 mmHg respectively) were calculated into AUCs using the Trapezoidal method.

b Number of subjects in whom the variable was obtained unless otherwise specified.

c Median (Q1-Q3).

d1 Two-tailed p-value of the median of the mean cumulative oxygen exposure in children with favorable outcome versus guideline normal range median: Wilcoxon signed-rank test.

d2 Two-tailed p-value of the median of the mean cumulative oxygen exposure in children with non-favorable outcome versus guideline normal range median: Wilcoxon signed-rank test.

Subgroup analyses are presented in Supplemental Tables S2-S6. No significant associations in multivariable logistic regression were found for a different definition of favorable neurologic outcome or arrest location (OHCA or IHCA). In the infant age group (<1 year old), cumulative PaCO_2_ exposure 0–24 h post-ROC was associated after adjustment with survival to hospital discharge (OR 0.80, 95 % CI 0.64–0.99).

### Adherence to post-cardiac arrest care guideline recommendations

Median cumulative PaO_2_ exposure during the three time periods was significantly higher than the recommended ranges as depicted in Table 3 (p < 0.001 for all). During the 0–6 h interval, PaCO_2_ exposure was lower than recommended ranges (Table 3, p < 0.001). Within 0–24 h post-ROC, cumulative PaO_2_ and PaCO_2_ remained within recommended ranges for 24 % and 58 % of children, respectively. Achieving both normoxia and normocapnia was observed in 12 % of children.

## Discussion

In this multicenter prospective cohort of children with cardiac arrest (with retrospective collected blood gas data), no association was found in multivariable analysis between cumulative (AUC) PaO_2_ and PaCO_2_ exposure in the first 24 h post-ROC and survival with favorable neurologic outcome. The AHA recommended post-cardiac arrest care normoxia and normocapnia targets were often not met, especially for normoxia. In 76 % of the population, children had cumulative hyperoxic PaO_2_ levels. The study's findings should be interpreted considering its limitations. While the AUC method can quantify fluctuations in oxygen and carbon dioxide levels, including time spent above and below recommended ranges, its accuracy is most optimal with structured sampling.

The trapezoidal AUC method has only been used twice in post-cardiac arrest care papers 12, 16. The Trapezoidal method, commonly used in pharmacokinetics for AUC calculation, can quantify PaO_2_ and PaCO_2_ fluctuations, but its accuracy depends on sample frequency and may not account for irregular sampling intervals 12, 15. For reliability, we included a minimum number of blood gas samples and analysed three time intervals within 24 h post-ROC to capture direct cell injury and reperfusion injury. However, the actual AUC values for PaO2 and PaCO2 are unknown, making it difficult to determine the number of individual measurements required for a reliable AUC approximation.. Moreover, ABG analysis remains intermittent, invasive and based on clinical indication.

Due to this methodological approach, our findings may be challenging to compare. A systematic review and *meta*-analysis revealed that most oxygenation and ventilation post-ROC target studies lack statistical significance and carry high bias risks ^13^. Children show more diverse underlying causes and higher respiratory issue prevalence than adults ^1^. This diversity may impede the determination of optimal oxygenation and ventilation strategies ^8^. Thus, the post-cardiac arrest care guideline recommendations rely more on logical merit than on empirical data ^8^.

Our findings indicate that children frequently experienced hyperoxia across all three time intervals and hypocapnia during the 0–6 h interval. Due to our multicentre design, our findings raise questions about adherence to the recommended post-cardiac arrest care normal ranges, particularly within the crucial first 6 h post-ROC. Despite these deviations, our study did not establish association with outcomes. The additional analysis to explore a potential U-shaped association yielded non-significant results. This does not preclude a U-shaped association curve, as the distribution is relatively skewed towards hyperoxic and hypercapnic values. Further investigation is required to confirm if maintaining normal ranges during these critical period indeed leads to reduced free radical formation or improved cerebral blood flow.

### Oxygenation

Our findings are consistent with those of van Zellem et al., demonstrating no association between cumulative oxygen levels and outcome ^12^. Notably, our study did not consider targeted temperature management as a confounder, as temperature control has become the accepted therapy instead of active cooling ^8^. During active cooling, oxygen pressure is lower as is carbon dioxide production (due to decreased metabolic rates), than during normothermia.

In other pediatric post-cardiac arrest cohorts, utilizing retrospective and cutoff definitions, hyperoxia is common, and no associations have been found with outcome 9, 10, 11. Subsequent recent large RCTs in adult post-ROC from OHCA found no difference between higher and lower PaO_2_ targets on death or hospital admission with disability or coma ^17^. In the BOX trial, patients were randomized 2.5 h post-arrest and a minimal difference of 6–11 mmHg was achieved over the first 48 h. The EXACT trial which assessed two different SpO_2_ targets initiated within 40 min after ROC and the first blood gas in the ICU, favoured the higher SpO_2_ target group. However, there was a minimal difference between the groups at the primary endpoint, survival to hospital discharge. Furthermore, fewer pre-ICU admission hypoxemic episodes (16.1 % vs. 31.3 %, p < 0.001) were observed in the high target group underscoring the significance of the measurement of the total burden of oxygen exposure and possibly of the significance of early hypoxic damage ^18^. The first pediatric RCT (Oxy-PICU), also highlighted the challenge of achieving a conservative oxygen target with a quarter of SpO_2_ values in goal range albeit demonstrating a small possible beneficial effect ^19^. Importantly, children post-cardiac arrest were excluded in this trial.

### Ventilation

In the prospective study of pIHCA by Castillo et al., both hypocapnia and hypercapnia immediately after ROC defined by cutoff were linked to worse survival to hospital discharge compared to normocapnia ^11^. In our cohort, hypocapnia (defined by cutoff, <30 mmHg) was more often encountered (14 % vs 35 % in our cohort) as was hypercapnia (>50 mmHg, 28 % vs 63 % in our cohort). Furthermore, significantly more patients with hypo- and hypercapnia defined by cutoff (<30 mmHg and > 50 mmHg) were found within the unfavorable outcome group (p < 0.001 for both).

The disparity in PaCO_2_ abnormality rate likely stems from methodological differences: in Castillo’s cohort, PaCO_2_ was determined based on one ABG directly post-ROC (versus our > 4 over 24 h) and OHCA cases, children often with ventilatory challenges, were excluded 3, 11.

In our subgroup analysis, higher cumulative PaCO_2_ exposure 0–24 h post-ROC in infants (< 1 year old) was associated with less survival to hospital discharge (OR 0.80, 95 % CI 0.64–0.99). This could imply that the infant brain is more susceptible to hypercapnia-induced vasodilation and warrants separate analysis. However, it's unknown if infants in this cohort also had a higher risk of intracerebral complications such as intraventricular haemorrhage due to vasodilation. It may also suggest that elevated PaCO_2_ serves as an indicator for a sicker, more difficult-to-ventilate child population. It's established that infant survival post-cardiac arrest is inferior compared to other age groups ^3^. Due to the limited sample size in subgroups, the results of the analyses could be an overestimation and should be interpreted as hypothesis generating.

The impact of carbon dioxide on cerebral autoregulation in hypoxic-ischemic encephalopathy remains uncertain, and whether modifying carbon dioxide levels can influence brain injury ^20^. The aforementioned *meta*-analysis based on two adult RCTs concluded that targeting moderate hypercapnia after cardiac arrest showed no significant effect on outcomes 13, 21, 22.

### Strengths and limitations

Strengths of the present study are the multicentre prospective design encompassing both OHCA and IHCA and the use of a cumulative PaO_2_ and PaCO_2_ exposure. However, this study also has several limitations regarding its generalizability. The inclusion criterion of at least 4 ABGs covering a minimum of 12 h post-ROC may introduce bias in two directions: it could favor the inclusion of more severely ill patients or lead to an overrepresentation of those with better outcomes. Specifically, children who experience shorter and more successful resuscitations may require fewer ABGs and might forgo the need for arterial lines altogether. Additionally, children who died early post-ROC may lack sufficient ABG samples, resulting in their exclusion and potentially skewing the cohort towards more favorable outcomes. We compared our findings with data from the broader pediRES-Q dataset, where survival rates in our cohort were indeed higher, highlighting these limitations further. Moreover, it is important to note that the results of the study are only valid for children who survive more than 12 h after ROC, as patients included in the study had ABGs spanning at least 12 h. Second, time measurements of ABG samples were not standardized. The more standardized measurements available independent of events, the more reliably the AUC can be calculated. The effect of short, severe peaks in PaO_2_ or PaCO_2_ that could trigger profound vasoconstriction or free oxygen radical forming could be underestimated. Third, in sensitivity analyses, the sample size of subgroups was small. This could result in underestimating the association with outcome since our covariables were preselected. Fourthly, some imported covariables were lacking such as pre- and intra-arrest PaO_2_ and PaCO_2_ levels, administered oxygen, ventilator settings and post-cardiac arrest care blood pressures. Lastly, while the PCPC score at discharge represents a more comprehensive outcome measure than mere survival, it may not suffice in assessing “true” neurological outcomes, especially in preschool-aged children ^14^.

### Future perspectives

In summary, cumulative PaO2 and PaCO2 exposure within the initial 24 h post-ROC didn't correlate with favorable neurological outcome in multivariable analysis, and post-cardiac arrest care normoxia and normocapnia goals weren't often achieved. Larger pediatric cohort studies are crucial to understand long-term effects and explore modulatory effects of other post-cardiac arrest care strategies in a bundles trial approach. Utilizing high-frequency data like the Oxygen Reserve Index (ORI) alongside conventional pulse oximetry shows promise for continuous, non-invasive monitoring within the mild hyperoxic range facilitating assessment of the actual abnormality “burden” (intensity and duration) ^23^. Controlled trials in adults reveal moderate target parameters, minimal group distinctions, and highly regulated trial protocols with little influence on clinical outcomes. ^24^. Future trials should focus on understanding underlying pathophysiology through measurement of brain injury, reactive oxygen species and cerebral blood flow ^24^.

### CRediT authorship contribution statement

**Marijn Albrecht:** Writing – original draft, Validation, Methodology, Investigation, Formal analysis, Data curation. **Rogier C.J. de Jonge:** Writing – review & editing, Supervision, Methodology, Conceptualization. **Jimena Del Castillo:** Writing – review & editing, Investigation, Data curation. **Andrea Christoff:** Writing – review & editing, Investigation, Data curation. **Matthijs De Hoog:** Writing – review & editing, Supervision. **Sangmo Je:** Writing – review & editing, Investigation, Data curation. **Vinay M. Nadkarni:** Writing – review & editing, Supervision. **Dana E. Niles:** Writing – review & editing, Investigation, Data curation. **Oliver Tegg:** Writing – review & editing, Investigation, Data curation. **Kari Wellnitz:** Writing – review & editing, Investigation, Data curation. **Corinne M.P. Buysse:** Writing – review & editing, Supervision, Methodology, Conceptualization.

## Declaration of competing interest

The authors declare the following financial interests/personal relationships which may be considered as potential competing interests: [Marijn Albrecht reports a relationship with ZOLL Medical Corporation that includes: funding grants. Vinay Nadkarni MD receives unrestricted grant funding to his institution from the National Institutes of Health, US Department of Defense, ZOLL Medical, Laerdal Foundation, RQI Partners, Philips Medical, and Nihon-Kohden. All are unrelated to this study. He serves as Editorial Board Member for Resuscitation and Pediatric Critical Care Medicine and was not involved in the editorial review or the decision to publish this article. Lastly, he serves on the Executive Committee for the Society of Critical Care Medicine (SCCM). The views expressed as an author in this manuscript are his, and are not intended to represent the views of the SCCM. Dana Niles was an employee of Children’s Hospital of Philadelphia at the time of data collection and analysis and is currently an employee of Philips Medical. If there are other authors, they declare that they have no known competing financial interests or personal relationships that could have appeared to influence the work reported in this paper].

## Appendix 1

PEDIATRIC RESUSCITATION QUALITY (pediRES-Q) COLLABORATIVE INVESTIGATORS^a^

Kamal Abulebda, Riley Hospital for Children, Indianapolis, IN, US; Diane Atkins, University of Iowa Stead Family Children’s Hospital, Iowa City, IA, US; Shilpa Balikai, University of Iowa Stead Family Children’s Hospital, Iowa City, IA, US; Marc Berg, Lucile Packard Children’s Hospital, Palo Alto, CA, US; Robert Berg, The Children’s Hospital of Philadelphia, Philadelphia, PA, US; Utpal Bhalala, Children’s Hospital of San Antonio, San Antonio, TX, US; Matthew S. Braga, Dartmouth-Hitchcock Medical Center, Lebanon, NH, US; Corinne Buysse, Erasmus MC– Sophia Children’s Hospital, Rotterdam, NL; Adam Cheng, Alberta Children’s Hospital, Calgary, AB, CA; Andrea Christoff, Children’s Hospital at Westmead, Sydney, NSW; Kelly Corbett, Dartmouth-Hitchcock Medical Center, Lebanon, NH, US; Allan DeCaen, Stollery Children’s Hospital, Edmonton, AB, CA; Katherine Daniels, Children’s Healthcare of Atlanta, Atlanta, GA, US; Gabry deJong, Erasmus MC–Sophia Children’s Hospital, Rotterdam, NL; Jimena del Castillo, Hospital Maternoinfantil Gregorio Marañón, Madrid, ES; Maya Dewan, Cincinnati Children’s Hospital Medical Center, Cincinnati, OH, US; Aaron Donoghue, The Children’s Hospital of Philadelphia, Philadelphia, PA, US; Ivie Esangbedo, Seattle Children’s Medical Center, Seattle, WA, US; Michael Flaherty, Massachusetts General Hospital, Boston, MA, US; Stuart Friess, St. Louis Children’s Hospital, St. Louis, MO, US; Sandeep Gangadharan, Mount Sinai Kravis Children’s Hospital, New York, NY, US; Orsola Gawronski, Ospedale Pediatrico Bambino Gesu‘, Rome, IT; Jonathan Gilleland, Alberta Children’s Hospital, Calgary, AB, CA; Heather Griffis, Healthcare Analytics Unit, The Children’s Hospital of Philadelphia, Philadelphia, PA, US; James Gray, Cincinnati Children’s Hospital Medical Center, Cincinnati, OH, US; Helen Harvey, Rady Children’s Hospital, San Diego, CA, US; Ilana Harwayne-Gidansky, Albany Medica Center, Albany, NY, US; Sarah Haskell, University of Iowa Stead Family Children’s Hospital, Iowa City, IA, US; Jennifer Hayes, Children’s Hospital of Orange County, Los Angeles, CA, US; Kiran Heber, Children’s Healthcare of Atlanta, Atlanta, GA, US; Betsy Hunt, Johns Hopkins University School of Medicine, Baltimore, MD, US; Takanari Ikeyama, Aichi Children’s Health and Medical Center, Obu, Aichi, JP; Priti Jani, The University of Chicago Medicine Comer Children’s Hospital, Chicago, IL, US; Monica Kleinman, Boston Children’s Hospital, Boston, MA, US; Lynda Knight, Lucile Packard Children’s Hospital Stanford, Palo Alto, CA, US; Hiroshi Kurosawa, Hyogo Prefectural Kobe Children’s Hospital, Kobe, Hyogo, JP; Kasper Glerup Lauridsen, The Children’s Hospital of Philadelphia, Philadelphia, PA, US; Aarhus University Hospital, Aarhus, DK; Tara Lemoine, Valley Children’s Hospital, Madera, CA, US; Tensing Maa, Nationwide Children’s Hospital, Columbus, OH, US; Elizabeth Masse, Seattle Children’s Hospital, Seattle, WA, US; Luz Marina Mejia, Instituto de Ortopedia Infantil Roosevelt, Bogota, CO; Yee Hui Mok, KK Women’s & Children’s Hospital, SG; Ryan Morgan, The Children’s Hospital of Philadelphia, Philadelphia, PA, US; Vinay Nadkarni, The Children’s Hospital of Philadelphia, Philadelphia, PA, US; Sholeen Nett, Dartmouth- Hitchcock Medical Center, Lebanon, NH, US; Dana Niles, The Children’s Hospital of Philadelphia, Philadelphia, PA, US; Amanda O’Halloran, The Children’s Hospital of Philadelphia, Philadelphia, PA, US; Michelle Olson, Children’s Hospital of Richmond, Richmond, VA, US; Gene Ong, KK Women’s & Children’s Hospital, SG; Prakad Rajapreyar, Children’s Hospital of Wisconsin, Milwaukee, WI, US; Tia Raymond, Medical City Children’s Hospital, Dallas, TX, US; Joan Roberts, Seattle Children’s Hospital, Seattle, WA, US; Anita Sen, NewYork-Presbyterian Morgan Stanley Children’s Hospital, New York, NY, US; Sophie Skellet, Great Ormond Street Hospital, London, UK; Daniel Stromberg, Dell Children’s Medical Center, Austin, TX, US; Felice Su, Lucile Packard Children’s Hospital Stanford, Palo Alto, CA, US; Robert Sutton, The Children’s Hospital of Philadelphia, Philadelphia, PA, US; Todd Sweberg, Cohen Children’s Medical Center, New Hyde Park, NY, US; Oscar Tegg, The Children’s Hospital at Westmead, Sydney, NSW; Ken Tegtmeyer, Cincinnati Children’s Hospital, Cincinnati, OH, US; Alexis Topjian, The Children’s Hospital of Philadelphia, Philadelphia, PA, US; Wendy Van Ittersum, Akron Children’s Hospital, Akron, OH, US; Javier Urbano Villaescusa; Hospital Maternoinfantil Gregorio Marañón, Madrid, ES; Ichiro Watanabe, Tokyo Metropolitan Children’s Hospital, Fuchu, Tokyo, JP; Denise Welsby, Great Ormond Street Hospital, London, UK; Jesse Wenger, Seattle Children’s Hospital, Seattle, WA, US; Heather Wolfe, The Children’s Hospital of Philadelphia, Philadelphia, PA, US; Andrea Yeo, National University Children’s Medical Institute, SG; Pricilla Yu, UT Southwestern Dallas Children’s Medical Center, Dallas, TX, US.
