# Supplementary material for: Association of cumulative oxygen and carbon dioxide levels with neurologic outcome after pediatric cardiac arrest resuscitation: A multicenter cohort study
Source: Resusc Plus. 2024 Oct 24;20:100804. doi: 10.1016/j.resplu.2024.100804 (PMC11541810; doi:10.1016/j.resplu.2024.100804)
Supplement: Supplementary Data 1 [file mmc1.pdf]

SUPPLEMENTARY FILES

1. IRB approval per site.

2. STROBE Checlist.

3. Supplemental Table 1. Univariable and multivariable logistic regression analysis of secondary outcomes: survival to hospital discharge and favorable neurologic outcome only in survivors (i.e. excluding PCPC 6).

4. Supplemental Table 2. Univariable and multivariable logistic regression analysis of all children with favorable (crude) neurologic outcome defined as survival with a PCPC of 1-2 or no difference with pre-arrest PCPC at discharge as dependent variable.

5. Supplemental Table 3. Univariable and multivariable logistic regression analysis of all children with survival to hospital discharge and favorable (crude) neurologic outcome defined as survival with a PCPC of 1-2 or no difference with pre-arrest PCPC at discharge as dependent variables split by location of arrest: out-of-hospital or in-hospital.

6. Supplemental Table 4. Univariable and multivariable logistic regression analysis of all children with survival to hospital discharge and favorable (crude) neurologic outcome defined as survival with a PCPC of 1-3 or no difference with pre-arrest PCPC at discharge as dependent variables split by location of arrest: out-of-hospital or in-hospital.

7. Supplemental Table 5. Univariable and multivariable logistic regression analysis of all children with survival to hospital discharge and favorable (crude) neurologic outcome defined as survival with a PCPC of 1-3 or no difference with pre-arrest PCPC at discharge as dependent variables split by age category: infant (< 1 year old), child (1-11 years old) and adolescent (12-17 years old).

8. Supplemental Table 6. Univariable and multivariable logistic regression analysis of all children with survival to hospital discharge and favorable (crude) neurologic outcome defined as survival with a PCPC of 1-3 or no difference with pre-arrest PCPC at discharge as dependent variables split by age category: infant (< 1 year old), child (1-11 years old) and adolescent (12-17 years old).

9. Supplemental Table 7. Univariable and multivariable logistic regression analysis of all children with survival to hospital discharge and favorable (crude) neurologic outcome defined as survival with a PCPC of 1-3 or no difference with pre-arrest PCPC at discharge as dependent variables split by age category: infant (< 1 year old), child (1-11 years old) and adolescent (12-17 years old).

IRB approval per site.

Site	IRB board name	Approval number	Approval date (dd-mmm-yy)	Study title
**Erasmus MC Sophia Children's Hospital** (Rotterdam, the Netherlands)	The Erasmus MC Medical Ethics Review Committee	MEC-2016-088	09th Feb. 2016	Quality of Pediatric Resuscitation in a Multicenter Collaborative: an Observational Study (pediRES-Q)
**The Hospital General Universitario Gregorio Marañón** (Madrid, Spain)	The Hospital General Universitario Gregorio Marañón Medical Ethics Review Committee	JCP-ADR-2016-01	20th Nov. 2017	Estudio Observacional Multicéntrico de la Calidad de la Reanimación Cardiopulmonar Pediátrica (pediRES-Q)
**The Children's Hospital at Westmead** (Syndey, Australia)	Sydney Children's Hospitals Network Human Research Ethics Committee	2019/ETH06002	17th Jan. 2019	Quality of Pediatric Resuscitation in a Multicenter Collaborative: an Observational Study (pediRES-Q)
**The Children's Hospital of Philadelphia** (Philadelphia, the United States)	The Children's Hospital of Philadelphia Research Institute IRB	15-012099	14th July 2015	Quality of Pediatric Resuscitation in a Multicenter Collaborative: an Observational Study (pediRES-Q)
**The University of Iowa Stead Family Children's Hospital** (Iowa, United States)	The Children's Hospital of Philadelphia Research Institute IRB	202104638	28th Jan. 2016	Quality of Pediatric Resuscitation in a Multicenter Collaborative: an Observational Study (pediRES-Q)

STROBE Statement

	Item No	Recommendation	Page No
**Title and abstract**	1	(*a*) Indicate the study's design with a commonly used term in the title or the abstract	1
		(*b*) Provide in the abstract an informative and balanced summary of what was done and what was found	1
		**Introduction**	
Background/rationale	2	Explain the scientific background and rationale for the investigation being reported	5
Objectives	3	State specific objectives, including any prespecified hypotheses	5
		**Methods**	
Study design	4	Present key elements of study design early in the paper	6
Setting	5	Describe the setting, locations, and relevant dates, including periods of recruitment, exposure, follow-up, and data collection	6
Participants	6	(*a*) Give the eligibility criteria, and the sources and methods of selection of participants. Describe methods of follow-up	6
		(*b*) For matched studies, give matching criteria and number of exposed and unexposed	NA
Variables	7	Clearly define all outcomes, exposures, predictors, potential confounders, and effect modifiers. Give diagnostic criteria, if applicable	7
Data sources/ measurement	8*	For each variable of interest, give sources of data and details of methods of assessment (measurement). Describe comparability of assessment methods if there is more than one group	6-7
Bias	9	Describe any efforts to address potential sources of bias	8
Study size	10	Explain how the study size was arrived at	6
Quantitative variables	11	Explain how quantitative variables were handled in the analyses. If applicable, describe which groupings were chosen and why	7
Statistical methods	12	(*a*) Describe all statistical methods, including those used to control for confounding	7-8
		(*b*) Describe any methods used to examine subgroups and interactions	7-8
		(*c*) Explain how missing data were addressed	7-8
		(*d*) If applicable, explain how loss to follow-up was addressed	7-8
		(*e*) Describe any sensitivity analyses	7-8
**Results**			

Participants	13*	(a) Report numbers of individuals at each stage of study—eg numbers potentially eligible, examined for eligibility, confirmed eligible, included in the study, completing follow-up, and analysed	10
		(b) Give reasons for non-participation at each stage	10
		(c) Consider use of a flow diagram	Fig1
Descriptive data	14*	(a) Give characteristics of study participants (eg demographic, clinical, social) and information on exposures and potential confounders	10
		(b) Indicate number of participants with missing data for each variable of interest	10
		(c) Summarise follow-up time (eg, average and total amount)	NA
Outcome data	15*	Report numbers of outcome events or summary measures over time	10
Main results	16	(*a*) Give unadjusted estimates and, if applicable, confounder-adjusted estimates and their precision (eg, 95% confidence interval). Make clear which confounders were adjusted for and why they were included	10-11
		(*b*) Report category boundaries when continuous variables were categorized	10-11
		(*c*) If relevant, consider translating estimates of relative risk into absolute risk for a meaningful time period	NA
Other analyses	17	Report other analyses done—eg analyses of subgroups and interactions, and sensitivity analyses	10-11
**Discussion**			
Key results	18	Summarise key results with reference to study objectives	12
Limitations	19	Discuss limitations of the study, taking into account sources of potential bias or imprecision. Discuss both direction and magnitude of any potential bias	15-16
Interpretation	20	Give a cautious overall interpretation of results considering objectives, limitations, multiplicity of analyses, results from similar studies, and other relevant evidence	12-15
Generalisability	21	Discuss the generalisability (external validity) of the study results	12-15
**Other information**			
Funding	22	Give the source of funding and the role of the funders for the present study and, if applicable, for the original study on which the present article is based	NA

*Give information separately for exposed and unexposed groups.

Supplemental Table 1. Univariable and multivariable logistic regression analysis of secondary outcomes: survival to hospital discharge and favorable neurologic outcome only in survivors (i.e. excluding PCPC 6).

	Survival to hospital discharge								Favorable neurologic outcome only in survivors (i.e. excluding PCPC 6)							
	Crude				Adjusted[a]				Crude				Adjusted[a]			
Variable	OR	95% CI		p-value	OR	95% CI		p-Value	OR	95% CI		p-value	OR	95% CI		p-Value
Area under the curve																
AUC PaO_2 0 - 6 h mmHg[b]	1.027	0.973	1.084	0.334	1.032	0.967	1.102	0.339	1.051	0.935	1.182	0.405	1.011	0.891	1.147	0.865
AUC PaO_2 7 - 24 h mmHg[b]	0.986	0.959	1.013	0.310	0.989	0.958	1.021	0.492	0.998	0.948	1.050	0.929	0.989	0.939	1.041	0.670
AUC PaO_2 0 - 24 h mmHg[b]	0.996	0.977	1.017	0.726	0.998	0.975	1.022	0.889	1.007	0.966	1.050	0.731	0.996	0.955	1.038	0.846
AUC $PaCO_2$ 0 - 6 h mmHg[b]	0.946	0.712	1.258	0.703	0.924	0.615	1.387	0.702	1.326	0.687	2.558	0.400	1.666	0.708	3.922	0.243
AUC $PaCO_2$ 7 - 24 h mmHg[b]	1.007	0.889	1.142	0.911	0.907	0.785	1.048	0.184	1.131	0.791	1.617	0.501	1.167	0.753	1.810	0.490
AUC $PaCO_2$ 0 - 24 h mmHg[b]	1.007	0.917	1.105	0.885	0.943	0.843	1.055	0.307	1.126	0.862	1.470	0.384	1.188	0.837	1.687	0.334

[a] Adjusted for: age at arrest, gender, location of arrest, rhythm, etiology of arrest, lowest pH within 24 hours and highest lactate within 24 hours.

[b] Value was rescaled by dividing by 100 in advance of the regression analysis.

Abbreviations: CI = Confidence Interval, Min. = Minimum, Max. = Maximum, Pa = Partial pressure, mmHg = Millimeter mercury, AUC = Area under the curve.

Supplemental Table 2. Univariable and multivariable logistic regression analysis of all children with favorable (crude) neurologic outcome defined as survival with a PCPC of 1-2 or no difference with pre-arrest PCPC at discharge as dependent variable.

	Survival with post-arrest PCPC 1-2 or ΔPCPC 0 at hospital discharge							
	Crude				Adjusted[a]			
Variable	OR	95% CI		p-value	OR	95% CI		p-value
<u>Area under the curve</u>								
AUC PaO_2 0 - 6 h mmHg[b]	1.043	0.988	1.100	0.125	1.046	0.980	1.117	0.172
AUC PaO_2 7 - 24 h mmHg[b]	1.002	0.974	1.03	0.905	1.006	0.974	1.038	0.725
AUC PaO_2 0 - 24 h mmHg[b]	1.008	0.987	1.028	0.461	1.010	0.986	1.035	0.405
AUC $PaCO_2$ 0 - 6 h mmHg[b]	0.959	0.720	1.278	0.776	1.017	0.679	1.523	0.935
AUC $PaCO_2$ 7 - 24 h mmHg[b]	1.042	0.919	1.181	0.521	0.976	0.847	1.125	0.738
AUC $PaCO_2$ 0 - 24 h mmHg[b]	1.028	0.937	1.128	0.561	0.996	0.891	1.114	0.944

[a] Adjusted for: age at arrest, gender, location of arrest, rhythm, etiology of arrest, lowest pH within 24 hours and highest lactate within 24 hours.

[b] Value was rescaled by dividing by 100 in advance of the regression analysis.

Abbreviations: CI = Confidence Interval, Min. = Minimum, Max. = Maximum, Pa = Partial pressure, mmHg = Millimeter mercury, AUC = Area under the curve, PCPC = Pediatric Cerebral Performance Category.

Supplemental Table 3. Univariable and multivariable logistic regression analysis of all children with survival to hospital discharge and favorable (crude) neurologic outcome defined as survival with a PCPC of 1-2 or no difference with pre-arrest PCPC at discharge as dependent variables split by location of arrest: out-of-hospital or in-hospital.

	Out-of-hospital cardiac arrest n = 133															
	Survival to hospital discharge								Survival with post-arrest PCPC 1-3 or ΔPCPC 0 at hospital discharge							
	Crude				Adjusted[a]				Crude				Adjusted[a]			
Variable	OR	95% CI		p-Value	OR	95% CI		p-Value	OR	95% CI		p-Value	OR	95% CI		p-Value
Area under the curve																
AUC PaO_2 0 - 6 h mmHg[b]	0.916	0.832	1.010	0.078	0.964	0.850	1.093	0.566	0.922	0.832	1.022	0.121	0.959	0.842	1.092	0.524
AUC PaO_2 7 - 24 h mmHg[b]	0.953	0.899	1.010	0.107	0.959	0.889	1.034	0.276	0.932	0.873	0.996	0.038	0.923	0.850	1.002	0.056
AUC PaO_2 0 - 24 h mmHg[b]	0.952	0.909	0.996	0.032	0.963	0.907	1.022	0.215	0.942	0.896	0.990	0.019	0.941	0.884	1.003	0.062
AUC $PaCO_2$ 0 - 6 h mmHg[b]	0.807	0.517	1.260	0.346	1.449	0.744	2.823	0.276	0.727	0.444	1.189	0.204	1.211	0.636	2.305	0.560
AUC $PaCO_2$ 7 - 24 h mmHg[b]	0.902	0.673	1.210	0.492	0.960	0.662	1.392	0.828	0.947	0.699	1.282	0.724	1.069	0.728	1.571	0.733
AUC $PaCO_2$ 0 - 24 h mmHg[b]	0.873	0.699	1.091	0.231	1.068	0.778	1.466	0.683	0.863	0.681	1.094	0.223	1.071	0.777	1.476	0.675

[a] Adjusted for: age at arrest, gender, rhythm, lowest pH within 24 hours and highest lactate within 24 hours.

[b] Value was rescaled by dividing by 100 in advance of the regression analysis.

Abbreviations: CI = Confidence Interval, Min. = Minimum, Max. = Maximum, Pa = Partial pressure, mmHg = Millimeter mercury, AUC = Area under the curve, PCPC = Pediatric Cerebral Performance Category.

Supplemental Table 4. Univariable and multivariable logistic regression analysis of all children with survival to hospital discharge and favorable (crude) neurologic outcome defined as survival with a PCPC of 1-3 or no difference with pre-arrest PCPC at discharge as dependent variables split by location of arrest: out-of-hospital or in-hospital.

	In-hospital cardiac arrest n = 155															
	Survival to hospital discharge								Survival with post-arrest PCPC 1-3 or ΔPCPC 0 at hospital discharge							
	Crude				Adjusted[a]				Crude				Adjusted[a]			
Variable	OR	95% CI		p-Value	OR	95% CI		p-Value	OR	95% CI		p-Value	OR	95% CI		p-Value
Area under the curve																
AUC PaO_2 0 - 6 h mmHg[b]	1.069	0.984	1.162	0.114	1.074	0.985	1.171	0.104	1.076	0.995	1.163	0.068	1.077	0.993	1.168	0.074
AUC PaO_2 7 - 24 h mmHg[b]	0.993	0.960	1.027	0.671	0.998	0.963	1.033	0.890	1.001	0.968	1.035	0.942	1.004	0.970	1.039	0.825
AUC PaO_2 0 - 24 h mmHg[b]	1.005	0.978	1.032	0.724	1.007	0.980	1.035	0.594	1.011	0.985	1.038	0.410	1.012	0.985	1.039	0.377
AUC $PaCO_2$ 0 - 6 h mmHg[b]	0.749	0.487	1.152	0.189	0.736	0.443	1.224	0.237	0.942	0.621	1.427	0.778	1.025	0.629	1.671	0.920
AUC $PaCO_2$ 7 - 24 h mmHg[b]	0.942	0.814	1.090	0.421	0.908	0.774	1.066	0.238	0.958	0.830	1.106	0.559	0.936	0.801	1.092	0.398
AUC $PaCO_2$ 0 - 24 h mmHg[b]	0.958	0.858	1.070	0.450	0.940	0.832	1.062	0.318	0.987	0.885	1.101	0.814	0.980	0.870	1.104	0.745

[a] Adjusted for: age at arrest, gender, rhythm, lowest pH within 24 hours and highest lactate within 24 hours.

[b] Value was rescaled by dividing by 100 in advance of the regression analysis.

Abbreviations: CI = Confidence Interval, Min. = Minimum, Max. = Maximum, Pa = Partial pressure, mmHg = Millimeter mercury, AUC = Area under the curve, PCPC = Pediatric Cerebral Performance Category.

Supplemental Table 5. Univariable and multivariable logistic regression analysis of all children with survival to hospital discharge and favorable (crude) neurologic outcome defined as survival with a PCPC of 1-3 or no difference with pre-arrest PCPC at discharge as dependent variables split by age category: infant (< 1 year old), child (1-11 years old) and adolescent (12-17 years old).

	Infant age group (< 1 year old) n = 111															
	Survival to hospital discharge								Survival with post-arrest PCPC 1-2 or ΔPCPC 0 at hospital discharge							
	Crude				Adjusted[a]				Crude				Adjusted[a]			
Variable	OR	95% CI		p-Value	OR	95% CI		p-Value	OR	95% CI		p-Value	OR	95% CI		p-Value
<u>Area under the curve</u>																
AUC PaO_2 0 - 6 h mmHg[b]	1.071	0.975	1.177	0.153	1.055	0.928	1.198	0.415	1.072	0.981	1.173	0.125	1.086	0.948	1.245	0.235
AUC PaO_2 7 - 24 h mmHg[b]	1.003	0.959	1.048	0.911	1.029	0.972	1.088	0.325	1.006	0.963	1.050	0.800	1.038	0.978	1.101	0.218
AUC PaO_2 0 - 24 h mmHg[b]	1.013	0.977	1.050	0.494	1.024	0.980	1.070	0.286	1.015	0.980	1.051	0.399	1.034	0.987	1.083	0.159
AUC $PaCO_2$ 0 - 6 h mmHg[b]	0.714	0.453	1.124	0.146	0.550	0.265	1.143	0.109	0.802	0.515	1.248	0.328	0.752	0.392	1.443	0.391
AUC $PaCO_2$ 7 - 24 h mmHg[b]	0.957	0.783	1.171	0.673	0.780	0.603	1.008	0.058	0.976	0.801	1.190	0.812	0.831	0.646	1.070	0.151
AUC $PaCO_2$ 0 - 24 h mmHg[b]	0.927	0.785	1.094	0.370	0.800	0.643	0.994	0.044	0.956	0.812	1.126	0.589	0.867	0.706	1.064	0.172

[a] Adjusted for: gender, location of arrest, age at arrest, rhythm, etiology of arrest, lowest pH within 24 hours and highest lactate within 24 hours.
[b] Value was rescaled by dividing by 100 in advance of the regression analysis.
Abbreviations: CI = Confidence Interval, Min. = Minimum, Max. = Maximum, Pa = Partial pressure, mmHg = Millimeter mercury, AUC = Area under the curve, PCPC = Pediatric Cerebral Performance Category.

Supplemental Table 6. Univariable and multivariable logistic regression analysis of all children with survival to hospital discharge and favorable (crude) neurologic outcome defined as survival with a PCPC of 1-3 or no difference with pre-arrest PCPC at discharge as dependent variables split by age category: infant (< 1 year old), child (1-11 years old) and adolescent (12-17 years old).

	Child age group (1 - 11 years old) n = 106															
	Survival to hospital discharge								Survival with post-arrest PCPC 1-2 or ΔPCPC 0 at hospital discharge							
	Crude				Adjusted[a]				Crude				Adjusted[a]			
Variable	OR	95% CI		p-Value	OR	95% CI		p-Value	OR	95% CI		p-Value	OR	95% CI		p-Value
Area under the curve																
AUC PaO_2 0 - 6 h mmHg[b]	0.935	0.850	1.028	0.164	0.998	0.882	1.128	0.971	0.967	0.879	1.063	0.481	1.029	0.913	1.160	0.638
AUC PaO_2 7 - 24 h mmHg[b]	0.981	0.937	1.027	0.402	0.985	0.934	1.038	0.568	0.977	0.931	1.025	0.344	0.979	0.926	1.034	0.445
AUC PaO_2 0 - 24 h mmHg[b]	0.978	0.944	1.013	0.209	0.989	0.948	1.031	0.593	0.982	0.947	1.018	0.314	0.991	0.949	1.034	0.666
AUC $PaCO_2$ 0 - 6 h mmHg[b]	1.101	0.644	1.884	0.725	1.671	0.685	4.075	0.259	1.167	0.674	2.020	0.582	1.780	0.744	4.259	0.195
AUC $PaCO_2$ 7 - 24 h mmHg[b]	1.114	0.878	1.414	0.373	1.129	0.827	1.542	0.444	1.112	0.889	1.390	0.352	1.115	0.851	1.460	0.429
AUC $PaCO_2$ 0 - 24 h mmHg[b]	1.072	0.913	1.258	0.395	1.136	0.869	1.485	0.351	1.082	0.923	1.268	0.331	1.134	0.889	1.445	0.311

[a] Adjusted for: gender, location of arrest, age at arrest, rhythm, etiology of arrest, lowest pH within 24 hours and highest lactate within 24 hours.

[b] Value was rescaled by dividing by 100 in advance of the regression analysis.

Abbreviations: CI = Confidence Interval, Min. = Minimum, Max. = Maximum, Pa = Partial pressure, mmHg = Millimeter mercury, AUC = Area under the curve, PCPC = Pediatric Cerebral Performance Category.

Supplemental Table 7. Univariable and multivariable logistic regression analysis of all children with survival to hospital discharge and favorable (crude) neurologic outcome defined as survival with a PCPC of 1-3 or no difference with pre-arrest PCPC at discharge as dependent variables split by age category: infant (< 1 year old), child (1-11 years old) and adolescent (12-17 years old).

	Adolescent age group (12 - 17 years old) n = 71															
	Survival to hospital discharge								Survival with post-arrest PCPC 1-2 or ΔPCPC 0 at hospital discharge							
	Crude				Adjusted[a]				Crude				Adjusted[a]			
Variable	OR	95% CI		p-Value	OR	95% CI		p-Value	OR	95% CI		p-Value	OR	95% CI		p-Value
Area under the curve																
AUC PaO_2 0 - 6 h mmHg[b]	1.084	0.967	1.216	0.165	1.133	0.909	1.412	0.268	1.073	0.963	1.196	0.201	1.060	0.874	1.287	0.554
AUC PaO_2 7 - 24 h mmHg[b]	0.970	0.909	1.036	0.368	0.935	0.850	1.028	0.164	0.983	0.922	1.049	0.611	0.972	0.885	1.068	0.556
AUC PaO_2 0 - 24 h mmHg[b]	1.002	0.961	1.046	0.910	0.987	0.924	1.054	0.697	1.006	0.965	1.050	0.767	0.995	0.931	1.063	0.885
AUC $PaCO_2$ 0 - 6 h mmHg[b]	1.052	0.609	1.817	0.857	1.313	0.470	3.667	0.604	1.084	0.627	1.875	0.772	1.273	0.471	3.437	0.634
AUC $PaCO_2$ 7 - 24 h mmHg[b]	0.687	0.444	1.062	0.091	0.447	0.185	1.079	0.073	0.760	0.497	1.162	0.205	0.512	0.217	1.209	0.127
AUC $PaCO_2$ 0 - 24 h mmHg[b]	0.910	0.716	1.156	0.438	0.873	0.581	1.313	0.515	0.941	0.740	1.196	0.618	0.887	0.584	1.347	0.574

[a] Adjusted for: gender, location of arrest, age at arrest, rhythm, etiology of arrest, lowest pH within 24 hours and highest lactate within 24 hours.

[b] Value was rescaled by dividing by 100 in advance of the regression analysis.

Abbreviations: CI = Confidence Interval, Min. = Minimum, Max. = Maximum, Pa = Partial pressure, mmHg = Millimeter mercury, AUC = Area under the curve, PCPC = Pediatric Cerebral Performance Category.
